# ﻿The new highest number of B chromosomes (Bs) in Leisler’s bat *Nyctalusleisleri* (Kuhl, 1817)

**DOI:** 10.3897/CompCytogen.v16i3.89911

**Published:** 2022-09-30

**Authors:** Marija Rajičić, Ivana Budinski, Milan Miljević, Branka Bajić, Milan Paunović, Mladen Vujošević, Jelena Blagojević

**Affiliations:** 1 Institute for Biological Research “Siniša Stanković” National Institute of the Republic of Serbia, Bulevar despota Stefana 142, 11040 Belgrade, Serbia Institute for Biological Research “Siniša Stanković” National Institute of the Republic of Serbia Belgrade Serbia; 2 Natural History Museum, Njegoševa 51, 11111 Belgrade, Serbia Natural History Museum Belgrade Serbia

**Keywords:** B chromosomes, Chiroptera, *Nyctalusleisleri*

## Abstract

B chromosomes (Bs) are supernumerary to the standard chromosome set, from which they prevalently derive. Variation in numbers both among individuals or populations and among cells within individuals is their constant feature. Leisler’s bat *Nyctalusleisleri* (Kuhl, 1817) is one of only four species of Chiroptera with detected Bs. Four males of *N.leisleri* were collected from two localities on the territory of Serbia and cytogenetically analysed. All animals had Bs with interindividual variability ranging from two to five heterochromatic micro Bs. The highest number of Bs was detected in this species. Among mammals, Rodentia and Chiroptera are orders with the largest number of species, but Bs frequently appear in rodents and rarely in chiropterans. Possible explanations for this difference are offered.

## ﻿Introduction

B chromosomes (Bs) are supernumerary but dispensable karyotype components of standard karyotypes (A chromosomes). Although their appearance has been known for more than a century, many questions related to them still seek answers. These additional elements are frequently present in different species of animals, plants, and fungi. It is estimated that 3% of all analysed species contain Bs (D’Ambrosio et al. 2017). Why they are frequently present in some species but not in others, and why are they absent or rare in specific taxa of animals and plants are among these riddles. Usually, Bs originate from A chromosomes of the same species, but also through hybridization between two closely related species (reviewed in Camacho et al. 2000; Jones and Houben 2003; Houben et al. 2014; Valente et al. 2016). They show significant variability in morphology, size, and number in which they appear in some species, populations, and even in different tissues of an individual. Usually, they do not follow Mendelian segregation law rules and also do not recombine with chromosomes of the A set, thus following their own evolutionary destiny (Jones 2018). Although dispensable chromosomes are often heterochromatic, many recent studies have shown that they are transcriptionally active and, most likely, contribute to the phenotypes of their carriers (summarized for mammals in Vujošević et al. 2018).

B chromosomes have been detected in 85 mammalian species (Vujošević et al. 2018), and recently another bat species was added to this list – *Megadermaspasma* (Linnaeus, 1758) (Volleth et al. 2021). With more than 1440 species (Simmons and Cirranello 2022), bats represent the second-largest mammalian group. To date, extra chromosomes were detected in only four bat species, three vespertilionids *Pipistrellustenuis* (Temminck, 1840) (Bhatnagar and Srivastava 1974), *Myotismacrodactylus* (Temminck, 1840) (Obara et al. 1976) *Nyctalusleisleri* (Volleth 1992), and one megadermatid *Megadermaspasma* (Volleth et al. 2021). Compared to rodents, which are the largest mammalian order and have 61 species with B chromosomes detected, the presence of B chromosomes in bats seems to be far less frequent event. Here we will present possible reasons for this occurrence.

Leisler’s bat *Nyctalusleisleri* (Kuhl, 1817) is a medium-sized bat distributed throughout Europe up to 57°N (Dietz and Kiefer 2016). Although a widespread species, it is considered rare almost everywhere except in Ireland (Boston et al. 2015). It is a typical woodland bat, and it shows a clear preference for mature forests in most of its distribution area. *N.leisleri* roosts mainly in tree holes, and it forages over the canopy, along forest trails, and over water bodies (Dietz and Kiefer 2016). Nursery colonies are usually in tree holes and contain 20–50 females. Females of this species give birth to 1–2 young during June, in Great Britain and Ireland only one, but in the rest of the areal usually two (Dietz and Kiefer 2016). This species hibernates in tree holes as well, and occasionally in buildings or underground sites (Dietz and Kiefer 2016; Juste and Paunović 2016). Leisler’s bat migrates over longer distances with regular seasonal movements between summer and winter habitats (Hutterer et al. 2005). *N.leisleri* has been recorded at seven localities in Serbia. Records consist of single individuals (mainly males) captured using mist-nets at species’ foraging grounds from July to September. There are no known roosts of this species in Serbia, and there is a lack of information on habitat use. Additionally, there is no evidence of the reproduction of Leisler’s bat in Serbia (Paunović et al. 2020).

Volleth (1992) analysed the karyotypes of *N.leisleri* and found 1, 2, and 3 B chromosomes (2n=44, NFa=50, NF=54 + 1-3Bs) in three males originating from Turkey, Germany, and Greece, respectively. Additionally, karyotypes of one more specimen, from Poland (Fedyk and Fedyk 1970) was conventionally stained and analysed, and probably contained 2n = 46, with two microchromosomes in the karyotype.

The modern view on Bs highlights their role in genome evolution as an extra genomic compartment with huge potential and still unknown biological significance, making Bs very interesting for research on different levels. Here we studied the presence of Bs and cytogenetic characteristics of karyotypes in *Nyctalusleisleri* in Serbia, for the first time.

## ﻿Materials and methods

### ﻿Ethics statement

Capturing and sampling was carried out under the permit provided by the Ministry of Environmental Protection of the Republic of Serbia (nos. 353-01-2814/2019-04; 353-01-195/2020-04). Animals were safely released immediately after sampling.

### ﻿Sampling

**Figure 1.**
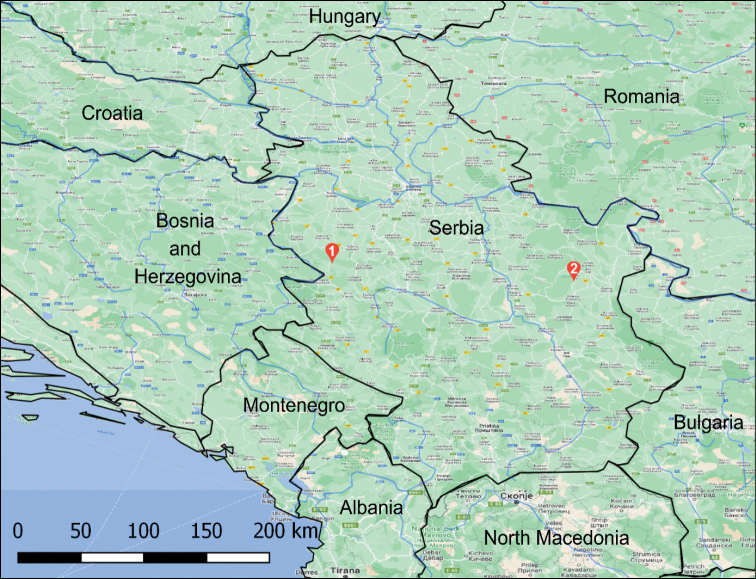
Map of sampling localities: 1. Bebića Luka, Western Serbia; 2. Zlot, Eastern Serbia.

Bats were captured at two localities in Serbia (Fig. 1): Bebića Luka (44.1963, 19.6962) in Western Serbia on 12.6.2020. and Zlot (44.0288, 21.9627) in Eastern Serbia on 1.9.2020. A total of four males of *Nyctalusleisleri* were captured.

Mist-nets were mounted over water bodies (river Jablanica and Lazareva river) before the sunset and remained open for 4 hours. All captured individuals were identified to the species level following Dietz and Kiefer (2016), sexed and age-determined based on the degree of ossification of the epiphyseal plates on finger bones (Brunet-Rossinni and Wilkinson 2009). Four adult males were captured (three in Bebića Luka and one in Zlot). Two tissue samples of plagiopatagium were taken from each individual using 3-mm sterile biopsy punch following Worthington and Barratt (1996) and immersed in physiological solution in the presence of antibiotics (penicillin 500000 U/l and kanamycin 500 mg/l) and antimycotic (amphotericin B 12,5 mg/l). Tissue samples were stored at 4 °C and transported to the laboratory within 24h from the moment of sampling.

### ﻿Cell culture

Primary fibroblast cell cultures were established using the protocol by Stanyon and Galleni (1991) and modified as in Romanenko et al. (2015). Cell passages were done each time when cells covered the flask surface completely. Dissociation of affixed cells was done by 0.25% trypsin, 0.2% EDTA. After a few passages the quantity of cells was sufficient for chromosome preparation.

Cells were kept in CO_2_ controlled incubator at 37 °C after adding colchicine (0.04 μg/ml) overnight and Ethidium bromide (EtBr 1.5 μg/ml) for three hours before cell picking. Cells were treated with hypotonic solution (33.5 mM KCl, 7.75 mM sodium citrate) and incubated for 55 minutes at 37 °C. Chromosomes were prefixed and fixed with fresh ice-cold fixative (methanol and glacial acetic acid in ratio 3:1). Slides for preparation were previously cleaned in chromic acid and well washed and preserved at 4 °C in distilled water.

### ﻿Chromosome preparations

Fibroblast cells grown in cell culture were used for chromosome preparations following the protocol described by Rajičić et al. (2017). One drop of chromosome suspension was spread on a slide and stained by Giemsa. The number of chromosomes was determined from at least 20 analysed metaphase plates per animal using Axias 2 plus (Zeiss) microscope. The standard chromosome complement of *N.leisleri* counts 44 chromosomes, and animals with more than 44 were considered to have Bs.

G-banding of metaphase chromosomes was performed according to the standard protocol (Graphodatsky and Radjabali 1988). Constitutive heterochromatin was detected by the modified techniques of C-banding (Sumner 1972). The position and number of nucleolus organizer regions (NORs) were identified using silver staining (Howell and Black 1980).

## ﻿Results

A total of four *N.leisleri* males were captured and their karyotypes were analysed by different cytogenetic methods for the first time in the territory of the Republic of Serbia. In three of the samples collected in Bebića Luka locality we detected the following karyotypes: one with 2n=44 +1-2Bs, two with 2n=44+3-5Bs, while the karyotype of the bat from Zlot had 2n=44+2-4Bs (Table 1).

**Table 1.** Intraindividual variability in number of B chromosomes in all studied samples. Number of cells with 0B, 1B, 2Bs, 3Bs, 4Bs, 5Bs and the total number of studied cells.

Sample	Locality	0B	1B	2Bs	3Bs	4Bs	5Bs	Total
**1**	Bebića Luka	0	**12**	**17**	2	0	0	31
**2**	Bebića Luka	0	1	3	6	**13**	6	29
**3**	Bebića Luka	0	1	0	0	7	**16**	24
**4**	Zlot	0	0	1	3	**16**	4	24

**Figure 2.**
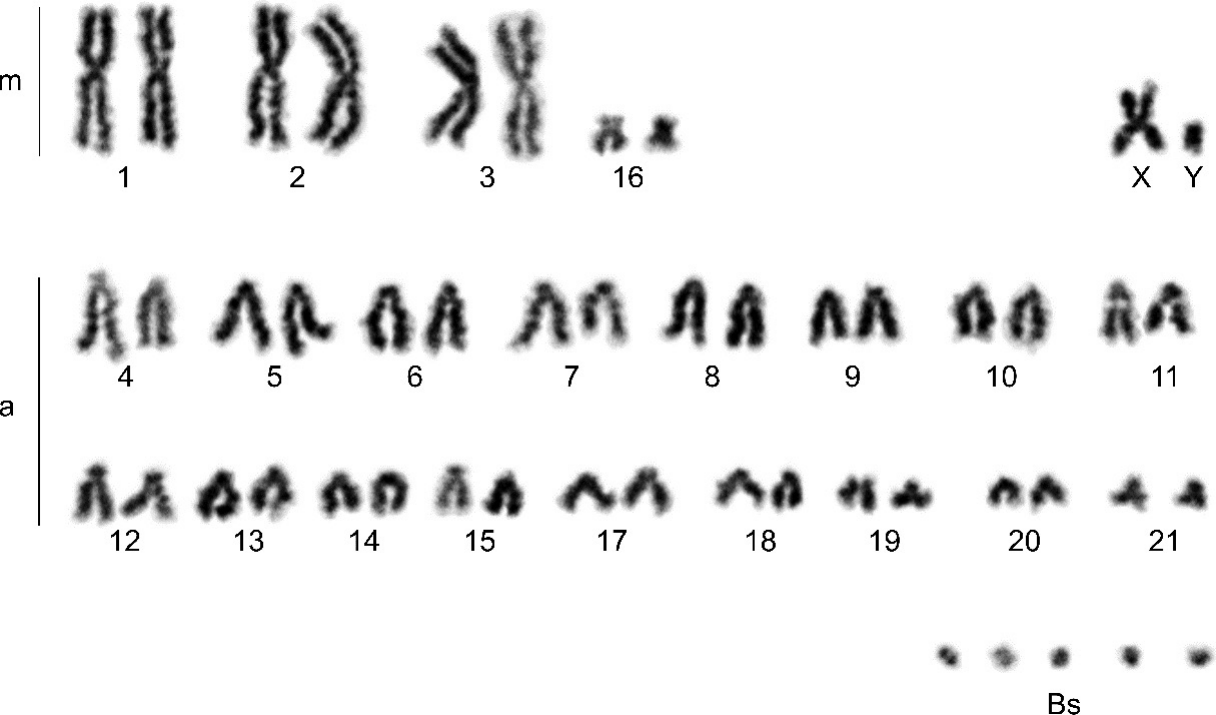
Conventionally stained karyotype of *N.leisleri* male with 5Bs (44+5Bs). m – metacentrics; a – acrocentrics; Bs – B chromosomes.

**Figure 3.**
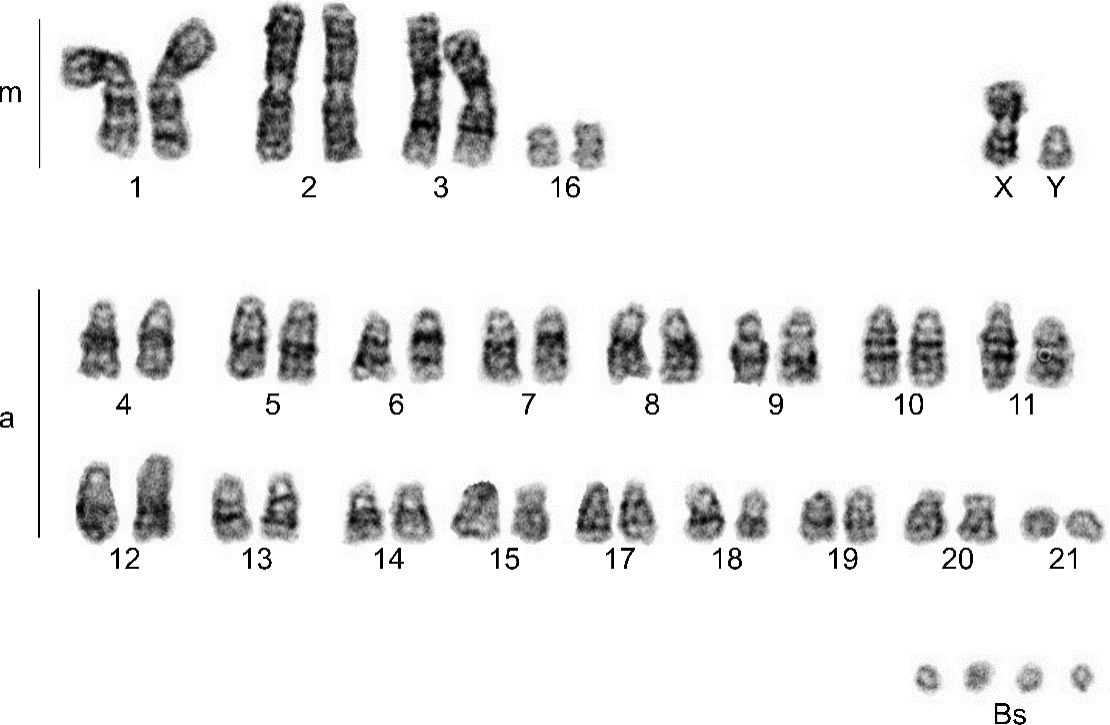
G-banded chromosomes of *N.leisleri* male with 4Bs (44+4Bs). m – metacentrics; a – acrocentrics; Bs – B chromosomes.

**Figure 4.**
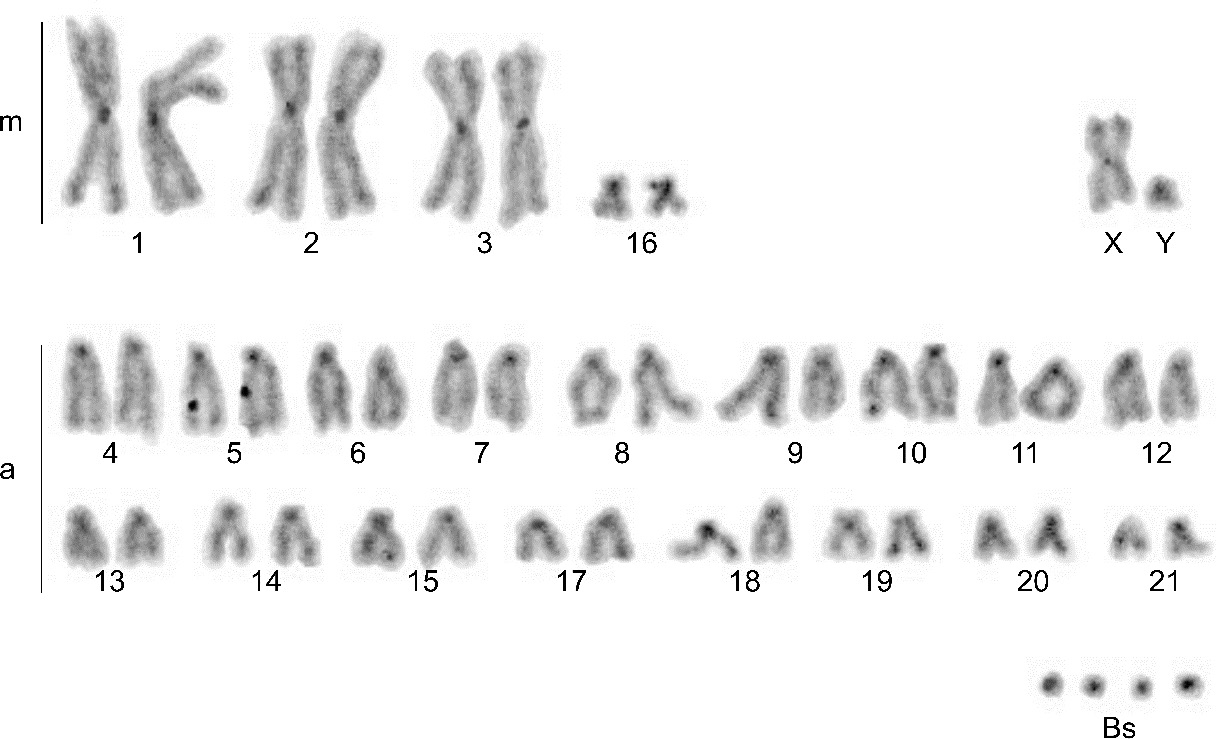
C-banded chromosomes of *N.leisleri* male with 4B (44+4Bs). m – metacentrics; a – acrocentrics; Bs – B chromosomes.

**Figure 5.**
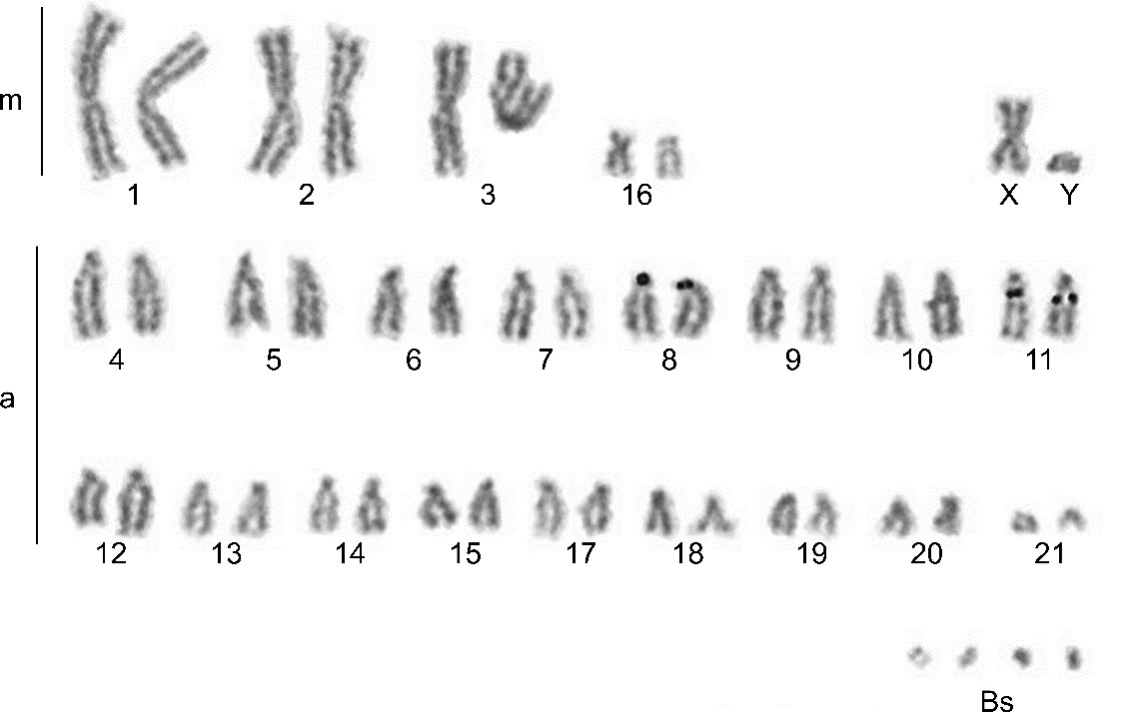
Nucleolus organiser regions (NORs) located on chromosome pairs no. 8 and 11 in *N.leisleri* male with 4Bs (44+4Bs). m – metacentrics; a – acrocentrics; Bs – B chromosomes.

Analysed karyotypes of all specimens consist of 42 autosomes, pair of sex chromosomes (XY) and variable number of Bs (2–5). Among the autosomes, three pairs were large metacentrics, one pair was small submetacentric, and the remaining 17 pairs were acrocentrics. The X chromosome was a medium-sized metacentric, and the Y chromosome was a small acrocentric. All Bs were microchromosomes (Figs 2–5).

C-banding showed the presence of constitutive heterochromatin in centromeric regions of all autosomes, sex chromosomes, and Bs (Fig. 4).

NORs were detected on two pairs of chromosomes in all analysed metaphases of all specimens from Serbia. In one pair, the active NORs were located on the minute arm of a pair of acrocentric chromosomes, while on the other pair, they were intercalary positioned, at the place of secondary constriction. Chromosomes are arranged from left to right and numerated in decreasing order, so the acrocentric pair with NORs at minute arms was at chromosome pair no. 8, and intercalary NORs were at pair no. 11 in the karyotype (Fig. 5).

## ﻿Discussion

After rodents, bats are the second most numerous group of mammals (Burgin et al. 2018). While rodents are the most frequent carriers of Bs, with 61 species possessing them, additional chromosomes are detected only in four bat species to date (Vujošević et al. 2018; Volleth et al. 2021). In vespertilionids, Bs are heterochromatic microchromosomes (reviewed Vujošević et al. 2018), while in *Megadermaspasma*, although among the smallest chromosomes, Bs are not microchromosomes and do not seem to be fully heterochromatic (Volleth et al. 2021). Least pipistrelle, *Pipistrellustenuis* (previously *Pipistrellusmimus*) has two or four metacentric Bs (Bhatnagar and Srivastava 1974). Big-footed Myotis, *Myotismacrodactylus*, possess one micro B chromosome that can be acrocentric or metacentric (Obara et al. 1976). It is known that the species *N.leisleri* contains heterochromatic micro B chromosomes in addition to the standard karyotype, but until now, the highest recorded Bs number was three (Volleth 1992). Our study is the first study of the *N.leisleri* karyotype in the territory of the Republic of Serbia. Previously published cytogenetic analyses (Volleth 1987) showed the same number but different positions of active nucleolus organisers (NORs) in this species. We obtained NORs at chromosome pairs 8 and 11 in *N.leisleri*, while according to Volleth (1987) they were on 8^th^ and 15^th^ chromosome pairs in specimens from Greece and Turkey. These differences could be a result of spatial diversity. However, we must not omit differences in the degree of chromosome condensation during preparation, which can be a problem when there are many acrocentrics of similar size in the karyotype. Furthermore, basic set in our samples consists of 44 chromosomes, 42 autosomes and pair of sex chromosomes, (2n=44, NFa=50, NF=54 + 2-5Bs) of the same morphology as it was previously described (Valleth 1992; Aslan and Zima 2014). Interestingly, all analysed *N.leisleri* samples were males and all of them got Bs in the karyotype (Volleth 1992; Aslan and Zima 2014). The only one female karyotype reported with 2n=46 from Poland (Fedyk and Fedyk 1970), seems to have two micro B chromosomes. Authors probably did not reported Bs since they analysed only one animal.

Bs are found in all major taxonomic groups of animals except birds (Vujošević and Blagojević 2004). However, recently, tissue-specific B-like chromosomes, restricted to germline cells (germline restricted chromosomes – GRCs), appeared to be widely present in songbird species (Torgasheva et al. 2019). As previously mentioned, Bs are found in only four species of bats. Small genome sizes characterize both birds and bats. Genome size in birds has a narrow range from 2 to 4 pg (Tiersch and Wachtel 1991). Furthermore, birds’ content of repeated sequences is the lowest among vertebrates (15–20%). A similar situation is characteristic for bats whose genome size is even smaller, averaging 2.35 pg (ranging from 1.3 to 3.2 pg) of DNA (Teeling et al. 2018). Bats are the only mammals capable of active flight and, together with birds, one of the two only living vertebrate taxa possessing this highly specialized mode of locomotion. It has been hypothesized that flight may impose a constraint on genome size. Genome size may be reduced in vertebrate groups having extreme metabolic demands for flight based on the relationship between genome size, cell size, and mass-specific metabolic rate (Hughes and Hughes 1995; Gregory 2002; Organ and Shedlock 2009). Smaller cells that characterize small genomes have a higher surface area to volume ratio, allowing improved gas exchange to satisfy metabolic demands (Szarski 1983). Reduced genome size may be why both birds and bats cannot tolerate the presence of Bs. Additionally, in flowering plants, the presence of Bs positively correlates with total genome size, and Bs frequently do not feature species with small genomes (Trivers et al. 2004).

The unique life-history traits of bats can also contribute to this non-acceptance of Bs. Longevity, slow reproductive rates, and small litters (Racey and Entwhistle 1999) make a chance of establishing and maintaining Bs much less possible than in rodents, which are characterized by a short life span, fast reproductive rates, and large litters (Promislow and Harvey 1990). Also, one must take into account the frequency of bat karyotype studies, compared to the ones conducted on rodents.

*Nyctalusleisleri* is considered to be a migratory species in Europe, generally following the NE-SW direction between summer roosts in Northeastern Europe and hibernation sites in central and southwestern parts of Europe (Hutterer et al. 2005; Boston et al. 2020). In other parts of Europe (NW and SE) this species may be vagrant or sedentary (Bogdanowicz and Ruprecht 2004), while data on the migration of *N.leisleri* in Eastern Europe is scarce (Hutterer et al. 2005). The longest migratory distances (over 1500 km) were recorded in females that bred in Germany and hibernate on the Iberian Peninsula (Ohlendorf et al. 2000; Wohlgemuth et al. 2004). According to Ruczyński (2004), males of Leisler’s bats occur sporadically in Poland and other northern regions but dominate in populations in Southern Europe. This is probably the reason why the only published karyotype of *N.leisleri* female is from Poland (Fedyk and Fedyk 1970). The vast majority of all captured Leisler’s bats in the territory of Serbia were males (Paunović et al. 2018; Paunović et al. 2020; Budinski unpublished data). There is no information on whether this species breeds in Serbia (Paunović et al. 2020). Scarce records of *N.leisleri* females in Serbia could be explained also by relatively low sampling efforts during the migration period.

The low number of analysed samples, the highest detected number of Bs, lack of data on female karyotype, and scarce data on this species’ ecology in the territory of Serbia, make *Nyctalusleisleri* very interesting model for further studies on Bs.
